# A Cyclam Salt as an Antifungal Agent: Interference with *Candida* spp. and *Cryptococcus neoformans* Mechanisms of Virulence

**DOI:** 10.3390/antibiotics13030222

**Published:** 2024-02-28

**Authors:** Fátima Cerqueira, Rui Medeiros, Inês Lopes, Carla Campos, Maria Pia Ferraz, Fernando Silva, Luís G. Alves, Eugénia Pinto

**Affiliations:** 1 FP-I3ID, FP-BHS, GIT-LoSa, University Fernando Pessoa, Praça 9 de Abril, 349, 4249-004 Porto, Portugal; fatimaf@ufp.edu.pt (F.C.); ruimedei@ipoporto.min-saude.pt (R.M.); 2 Faculty of Health Sciences, University Fernando Pessoa, Rua Carlos da Maia, 296, 4200-150 Porto, Portugal; 3 Molecular Oncology and Viral Pathology Group, Research Center of IPO Porto (CI-IPOP)/RISE@CI-IPOP (Health Research Network), Portuguese Oncology Institute of Porto (IPO Porto)/Porto Comprehensive Cancer Center (Porto.CCC) Raquel Seruca, Rua Dr. António Bernardino de Almeida, 4200-072 Porto, Portugal; 10170389@ess.ipp.pt (I.L.); carla.campos@ipoporto.min-saude.pt (C.C.); 4 School of Health, Polytechnic Institute of Porto, Rua Dr. António Bernardino de Almeida, 400, 4200-072 Porto, Portugal; 5 Department of Metallurgical and Materials Engineering, Faculty of Engineering (FEUP), University of Porto (UP), 4200-465 Porto, Portugal; mpferraz@fe.up.pt; 6 i3S—Institute for Research and Innovation in Health, University of Porto (UP), 4099-002 Porto, Portugal; 7 Institute of Biomedical Engineering (INEB), University of Porto (UP), 4099-002 Porto, Portugal; 8 Department of Pharmaceutical Technology, Faculty of Pharmacy, University of Coimbra, 3000-548 Coimbra, Portugal; fernando_silva9@hotmail.com; 9 REQUIMTE/LAQV, Group of Pharmaceutical Technology, Faculty of Pharmacy, University of Coimbra, 3000-548 Coimbra, Portugal; 10 Centro de Química Estrutural—Institute of Molecular Sciences, Associação do Instituto Superior Técnico para a Investigação e Desenvolvimento, Av. António José de Almeida nº12, 1000-043 Lisboa, Portugal; luis.g.alves@tecnico.ulisboa.pt; 11 Laboratory of Microbiology, Biological Sciences Department, Faculty of Pharmacy, University of Porto (UP), Rua Jorge de Viterbo Ferreira 228, 4050-313 Porto, Portugal; 12 CIIMAR/CIMAR, Interdisciplinary Centre of Marine and Environmental Research, Terminal de Cruzeiros do Porto de Leixões, 4450-208 Matosinhos, Portugal

**Keywords:** *Candida* spp., *Cryptococcus neoformans*, yeasts, virulence mechanisms, biofilms, cyclam salt

## Abstract

The importance of fungal infections, particularly those caused by yeasts, is increasing among the medical community. *Candida albicans* and *Cryptococcus neoformans* are amongst the high-priority fungal species identified by the World Health Organization (WHO) and are considered in the critical group, while *Candida krusei* is included in the medium-priority group. The cyclam salt H_4_[H_2_(^4-CF3^PhCH_2_)_2_Cyclam]Cl_4_ proved to be active against the growth of these three yeasts, and the aim of this work was to verify its interference with their virulence mechanisms, whether shared or unique. H_4_[H_2_(^4-CF3^PhCH_2_)_2_Cyclam]Cl_4_ significantly inhibited biofilm production and catalase activity, being able to interfere with *C. albicans* dimorphic transition and *C. neoformans* melanin production. At the minimal inhibitory concentration (MIC) values, H_4_[H_2_(^4-CF3^PhCH_2_)_2_Cyclam]Cl_4_ had no antioxidant effect, as determined by the DPPH method. When using the RAW264.7 macrophage cell line, H_4_[H_2_(^4-CF3^PhCH_2_)_2_Cyclam]Cl_4_ reduced nitric oxide (NO) detection (the Griess reaction), but this effect was associated with a significant toxic effect on the cells.

## 1. Introduction

**Figure 1:**
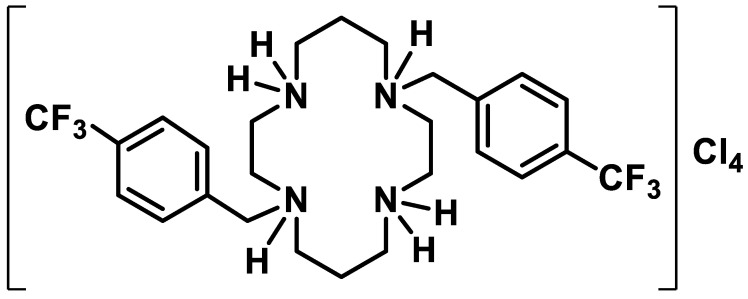
Chemical structure of H_4_[H_2_(^4-CF3^PhCH_2_)_2_Cyclam]Cl_4_ (**1**).

Cyclams are macrocyclic polyamines, the medical interest in which was fueled by the therapeutic potential of a bicyclam derivative against HIV infection, inflammatory diseases, cancer, and stem-cell mobilization [1]. Taking advantage of their biocompatibility, high metal chelation stability constants, and the possibility of N-functionalization of the cyclam backbone, a variety of cyclam derivatives have been successfully explored in recent years as antibacterial [2,3,4,5,6,7,8], antifungal [7,8,9], and antiparasitic agents [10,11,12]. The neamine cyclam derivative (NeaCyclam) was revealed to be highly effective against *Escherichia coli* and *Enterococcus aerogenes*, being able to affect the outer membrane stability by altering the permeability barrier [2]. *Trans*-disubstituted cyclams displaying naphthalimide, naphthalene-based, or single aromatic groups linked through 1,2,3-triazoles or simple alkyl chains were studied against *Mycobacterium tuberculosis* [3,4]. It was observed that replacing naphthalimide with naphthyl or benzyl groups led to a decrease in antibacterial activity. Importantly, the inhibitory effect was maintained against clinical isolates of *M. tuberculosis* resistant to single or multiple antimycobacterial drugs. A series of *trans*-disubstituted cyclam salts displaying benzyl groups directly attached to the cyclam ring was found to be active against *E. coli* and *Staphylococcus aureus* [6,7]. The results obtained revealed that the position and polarity of the substituents on the benzyl groups attached to the cyclam ring are directly related to the antibacterial activity, with the compound displaying a CF_3_ moiety in the *para* position of the benzyl groups being remarkably active. In addition, H_4_[H_2_(^4-CF3^PhCH_2_)_2_Cyclam]Cl_4_ (Figure 1) also displayed antifungal activity against *Candida albicans, Candida krusei,* and *Cryptococcus neoformans* [9]. The minimum inhibitory concentration (MIC) and minimum lethal concentration (MLC) values obtained for *C. albicans* and *C. krusei* were MIC = MLC = 128 and 8 μg/mL, respectively. *C. neoformans* displayed MIC = 4 μg/mL and MLC = 8 μg/mL.

*C. albicans*, *C. krusei*, and *C. neoformans* are all able to form biofilms, defined as three-dimensional organized structures constituted by clusters of microorganisms (bacteria or fungi) adhered in either biotic (e.g., host tissue) or abiotic (e.g., inert material) surfaces or interfaces and encased in a protective self-produced hydrated extracellular matrix composed mainly of proteins, polysaccharides, and extracellular DNA [13]. Although biofilm formation in vivo usually involves a combination of bacterial and fungal species, it is important to note that a single microbial species can also create a biofilm [14,15]. Since most microorganisms live in biofilms (about 80%), it is an essential area of research, with several studies focused on the destabilization of this structure [16,17]. Besides the production of biofilm, *C. albicans*, *C. krusei*, and *C. neoformans* also have the catalase enzyme that allows them to degrade hydrogen peroxide (H_2_O_2_) produced by immune cells [18,19,20]. Therefore, catalase inactivation renders yeasts extremely vulnerable to the deleterious effects of reactive oxygen species (ROS).

*C. albicans* is one of the most pathogenic yeasts for humans, causing superficial, systemic, and opportunistic infections. It is associated with high morbidity and mortality rates, and its pattern of antifungal resistance is of great importance [21]. The list of *C. albicans* virulence factors is extensive and includes its capacity for dimorphic transition [22]. *C. krusei*, sometimes neglected with respect to *C. albicans,* is intrinsically resistant to fluconazole and is of clinical interest too [21,23]. *C. neoformans* is a yeast that accounts for a great number of central nervous system infections, namely in diabetic and immunosuppressed patients. Some of its virulence factors include the presence of a capsule that allows it to escape from the immune system, the urease enzyme that facilitates yeast adhesion and brain invasion, and melanin production as a form of protection against ROS produced by host cells, among others [24].

Besides the virulence factors described above, the ability of these yeasts to acquire antifungal resistance mechanisms is one of the leading reasons for the search for new antifungal drugs, whether from plant extracts or natural or synthetic compounds [25,26,27].

Fungal infection triggers signaling cascades in the host that culminate in inflammation, which allows for the identification and elimination of the invading microorganism [28]. Inflammation is a host mechanism of defense against infection; since a few years ago, some of the commonly used antifungal agents were described as possessing modulatory effects, either increasing or inhibiting inflammatory processes [29,30,31]. Phagocytosis is one of the first lines of defense in the fight against fungi, and macrophages are the key cells that perform this defense. Nitric oxide (NO) produced by activated macrophages is thus a marker of inflammation [32,33]. Although the main objective of the immune system is the elimination of microorganisms, in some cases, this pro-inflammatory state can trigger deleterious processes, resulting in symptoms such as redness, itching, and swelling [34] and inflammation of the central nervous system [35].

In the present work, we studied the effect of H_4_[H_2_(^4-CF3^PhCH_2_)_2_Cyclam]Cl_4_ on the virulence mechanisms of *C. albicans*, *C. krusei*, and *C. neoformans* and explored its effect on NO production by a lipopolysaccharide (LPS)-stimulated RAW264.7 macrophage cell line. H_4_[H_2_(^4-CF3^PhCH_2_)_2_Cyclam]Cl_4_ inhibited biofilm formation and catalase activity. Concerning unshared yeast virulence factors, H_4_[H_2_(^4-CF3^PhCH_2_)_2_Cyclam]Cl_4_ inhibited *C. albicans* dimorphic transition and *C. neoformans* melanin production.

## 2. Results

### 2.1. Antibiofilm Activity

**Figure 2:**
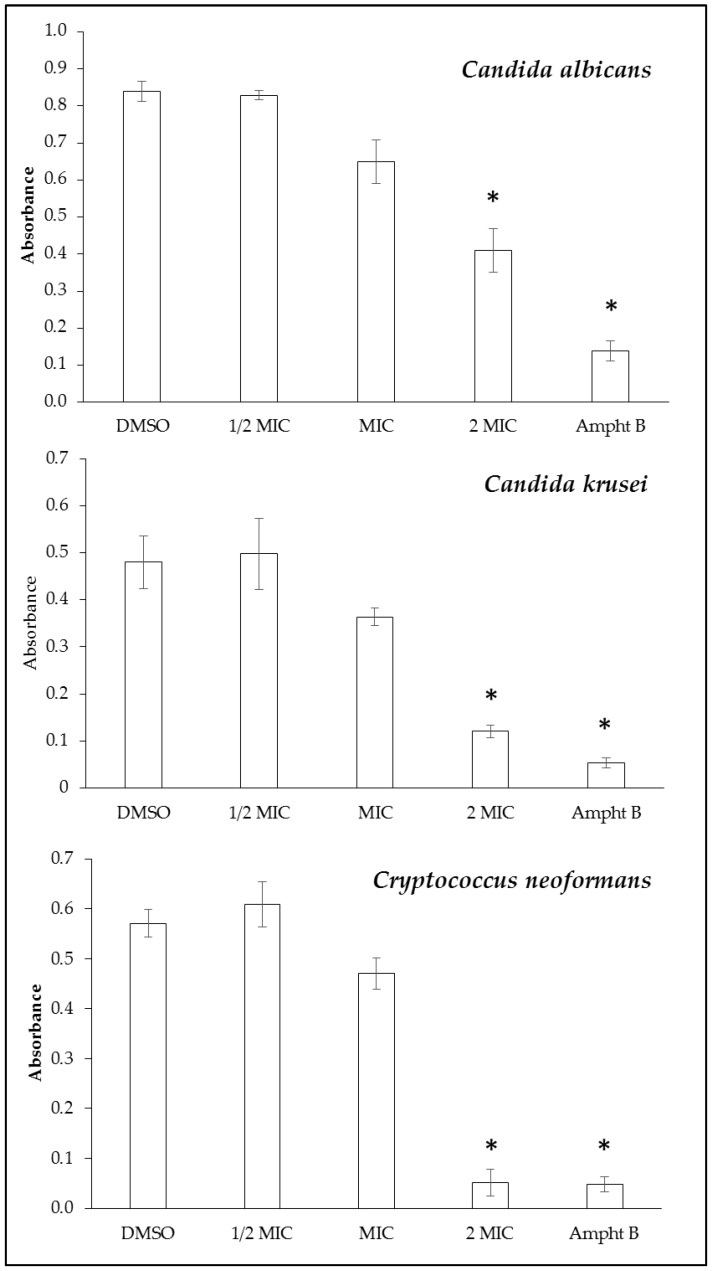
Antibiofilm activity of compound **1** against *C. albicans*, *C. krusei*, and *C. neoformans* at different concentrations (½ MIC, MIC, and 2 MIC) as well as the effect of solvent control (DMSO) and amphotericin B. DMSO, dimethylsulfoxide; MIC, minimal inhibitory concentration; Ampht B, amphotericin B. Three independent experiments were performed, and the results are shown as mean ± SD. * Mean significant differences (*p* < 0.05) between groups.

The antibiofilm activity of H_4_[H_2_(^4-CF3^PhCH_2_)_2_Cyclam]Cl_4_, **1**, was assessed on *C. albicans*, *C. krusei*, and *C. neoformans*, and the results are depicted in Figure 2. Compound **1** was able to inhibit the biofilm formation of *C. albicans*, *C. krusei*, and *C. neoformans* at concentration values corresponding to 2 MIC (MIC = 128, 8, and 4 μg/mL, respectively, [9]), showing a higher antibiofilm capacity for *C. krusei* and *C. neoformans*.

### 2.2. Interference with Catalase Activity

Catalase is an important enzyme for yeasts, since it protects them against the deleterious effects of H_2_O_2_ produced by host immune cells.

**Figure 3:**
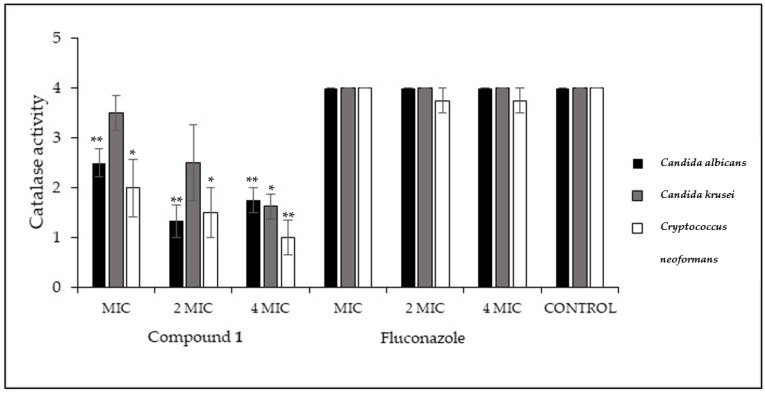
Effect of compound **1** in the catalase activity of *C. albicans*, *C. krusei*, and *C. neoformans* at different concentrations (MIC, 2 MIC, and 4 MIC) as well as the effect of fluconazole, serving as control of the experiment. A score for the visual effervescence observed (Y axis) was made, with (4) corresponding to maximum effervescence of the positive control (yeasts with DMSO but without compound **1**), (3) corresponding to 75%, (2) corresponding to 50%, and (1) corresponding to 25% of the effervescency of control. Three independent experiments were performed, and results are presented as mean ± SD. * *p* < 0.05; ** *p* < 0.01.

The effect of compound **1** on the catalase activity of *C. abicans*, *C. krusei*, and *C. neoformans* was evaluated, and the results are depicted in Figure 3.

Compound **1** significantly inhibited the catalase activity of *C. albicans* and *C. neoformans* for all the concentrations tested. On the other hand, the catalase activity of *C. krusei* was only significantly inhibited at concentrations as high as 4 MIC.

### 2.3. Inhibition of Candida albicans Dimorphic Transition

**Figure 4:**
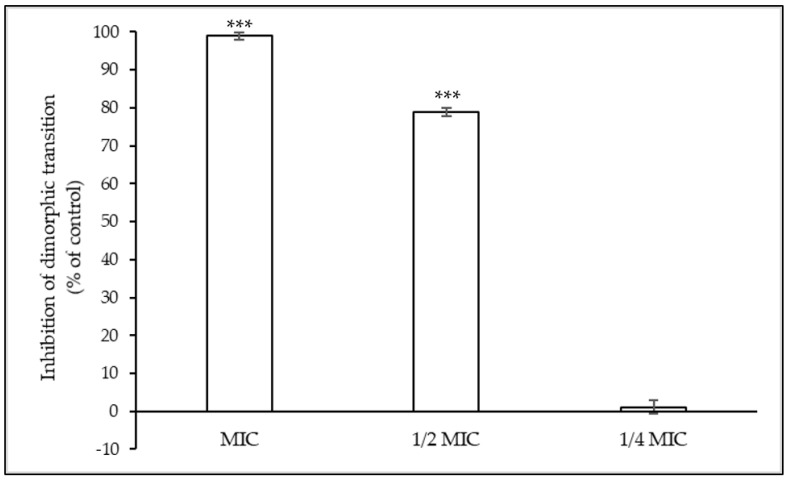
Effect of compound **1** on *Candida albicans* dimorphic transition at different concentrations (MIC, 1/2 MIC, and 1/4 MIC). Three independent experiments were performed, and the results are presented as mean ± SD. *** *p* < 0.001.

The effect of compound **1** on the dimorphic transition of *C. albicans* was evaluated at concentrations equal to or below MIC values (Figure 4).

Compound **1** significantly inhibited the dimorphic transition of *C. albicans* at MIC values when compared to the untreated control, which was considered as 100%. This inhibition was still significant (around 80%) at concentrations of 1/2 MIC.

### 2.4. Effect on Cryptococcus neoformans Urease

The production and activity of the urease enzyme produced by *C. neoformans* was not affected by treatment with compound **1** at concentration values of ½ MIC and ¼ MIC, as determined by visual observation of the coloration of the urease media after inoculation with the yeast.

### 2.5. Effect on Cryptococcus neoformans Melanin Production

*C. neoformans* possesses an enzyme that can produce melanin using caffeic acid as a substrate to protect it against ROS generated by the host cells. The ability of compound **1** to interfere with melanin production, a virulence factor of *C. neoformans*, was evaluated, and the results are presented in Table 1.

Compound **1** significantly inhibited the melanin production by *C. neoformans* at 1/8 MIC, which was easily detected by visual observation. 

### 2.6. Antioxidant Effect

**Figure 5:**
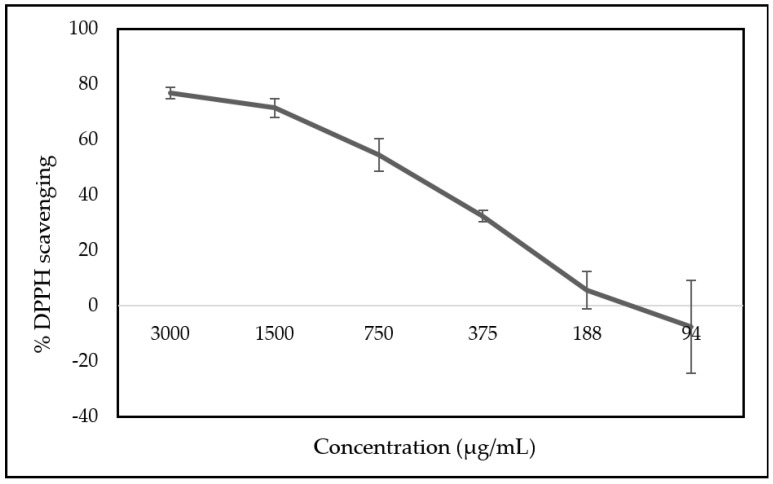
Percentage of DPPH scavenging according to the concentration of compound **1**. Three independent experiments were performed, and the results are presented as mean ± SD. DPPH: 2,2-diphenyl-1-picyryl hydrazil.

The ability to scavenge free radicals in comparison to the stable radical 2,2-diphenyl-1-picyryl hydrazil (DPPH) was initially chosen because it is a simple, fast, and sensitive methodology. The correlation of the antioxidant effect with the concentration of compound **1** revealed an almost linear trend, as can be observed in Figure 5. The results highlight that compound **1** displayed an antioxidant capacity of >40% at higher concentrations (3000 to 750 µg/mL), which was lost at MIC values (Figure 5). No radical scavenging was detected for concentrations below 188 µg/mL despite the range of concentrations tested (3000 to 1 µg/mL). For this reason, Figure 5 only represents the range of concentrations where the DPPH radical scavenging occurred.

### 2.7. Effect on NO Production by LPS-Stimulated RAW264.7 Macrophages

Nitric oxide (NO) is produced by macrophages as a marker of activation and inflammation. The effect of compound **1** on NO production by LPS-stimulated RAW264.7 murine macrophages was evaluated (Table 2).

Compound **1** could significantly decrease the production of NO by the LPS-stimulated murine macrophage cell line RAW264.7 at concentrations of 64 and 32 μg/mL without affecting the macrophage viability. For concentrations ≥128 μg/mL, the inhibition of NO production was followed by an important decrease in the macrophages’ metabolic viability. These results allowed us to conclude that, at 64 and 32 μg/mL, compound **1** reduced the NO production by the macrophages and that this inhibition was not due to cytotoxicity. The production of NO was not affected by compound **1** at a concentration of 16 μg/mL, which was higher than the MIC values for *C. krusei* (8 µg/mL) and *C. neoformans* (4 µg/mL). 

## 3. Discussion

Fungal infections are increasingly becoming a problem that needs to be tackled, not only because of the growing virulence of the strains but also because of the growing resistance to the antifungal drugs available for therapy. The World Health Organization (WHO) recently created the “WHO fungal priority pathogens list to guide research, development, and public health action”, a list of priority fungi classified according to the degree of threat to public health and the risk of antifungal resistance [36]. According to these assumptions, the fungi were classified into three categories: critical, high, and medium priority, with *C. neoformans* and *C. albicans* being considered in the critical group, while *C. krusei* is in the medium group [36]. In this sense, the authors consider the search for new active compounds against these fungi to be extremely important, leading to the development of the research work presented here.

The development of biofilms by yeasts is one of the important factors in the development of resistance to antifungal agents [36]. As shown in this study, compound **1** was able to inhibit the initial biofilm formation by *C. neoformans*, *C. albicans*, and *C. krusei*, which may present an advantage in the treatment of infections by these fungi, particularly in combination with antifungals already used in therapy.

Another virulence mechanism, common to all the yeasts studied, is the production of the enzyme catalase. Catalase enables yeasts to fight against stress response and phagocytic destruction [37]. Compound **1** significantly inhibited the catalase activity for all the yeasts under investigation.

*C. albicans* is part of the human microbiota, and dimorphic transition is a determinant of its virulence and pathogenicity [37]. The change from yeast to a hyphal-like morphology allows it to establish infection and invade the host tissues. Compound **1** inhibited the dimorphic transition at concentrations lower than the MIC value and, thus, it can reduce *C. albicans* virulence. The dimorphic transition is also involved in biofilm formation, and the inhibition of this filamentous formation could help and justify the inhibition of biofilm formation.

Urease is an enzyme that hydrolyses urea into carbon dioxide and ammonia, the latter increasing pH and allowing yeasts to defend themselves from phagocytosis as well as being a facilitator of brain invasion [24,38]. Compound **1** was not able to inhibit urease activity.

*C. neoformans* produces melanin for protection against ROS produced by immune cells. Melanin production is an important *C. neoformans* virulence factor, as it allows this yeast to escape from degradation after being phagocyted by macrophages [39]. In a medium with caffeic acid, *C. neoformans* uses this substrate to produce a brown pigment [40]. Some authors state that melanin-producing strains are more virulent than non-melanin-producing ones, and the capacity of producing melanin from catecholamines seems to be linked to *C. neoformans* neurotropism [24,41]. Compound **1** strongly reduced melanin production, which could have implications for *C. neoformans* neuroinvasion and escape from immune defense systems. This reduction in melanin production might achieve a higher importance if we consider that the increase in pH caused by urease intensifies melanization and invasion of the central nervous system [38]. 

When a fungal infection is caused by yeasts such as *Candida* spp. or *Cryptococcus* spp. an immune response is activated, and the first line of defense is based on the action of phagocytes to eliminate the invading microorganisms [42]. One of the phagocytic cells involved in this defense is macrophages, which produce reactive oxygen species, namely NO [42]. Compound **1** showed no antioxidant activity at MIC values, and no significant changes were observed concerning NO production at concentrations ≤16 μg/mL. Above 128 μg/mL, the inhibition of NO production was correlated with the loss of viability of the RAW254.7 macrophages. This means that for the MIC values range of *C. neoformans* and *C. krusei*, compound **1** does not affect NO production by LPS-stimulated RAW264.7 macrophages. It is worth mentioning that compound **1** significantly reduced the macrophage viability at MIC values of *C. albicans*.

## 4. Materials and Methods

### 4.1. Reagents, Standards, and Compounds

#### 4.1.1. Culture Media and Reagents Used in Yeasts Assays

Sabouraud dextrose broth (SDB) and urea media from Bio-Mèrieux (Marcy L’Etoile, France); yeast nitrogen base (YNB) (Becton, Dickinson and Company, Pont-de-Claix, France); phosphate-buffered saline (PBS) and Fisher’s reagent (Geel, Belgium); dimethyl sulfoxide (DMSO), N-acetylglucosamine (GlcNAc), proline, amphotericin B, fluconazole, 3-(N-morpholino) propanesulfonic acid (MOPS), caffeic acid (CA), and phenol red from Sigma-Aldrich (St. Louis, MO, USA); peptone from LiofilChem (Roseto degli Abruzzi, Italy); glucose and sodium chloride (NaCl) from Labkem (Barcelona, Spain); disodium phosphate and potassium dihydrogen phosphate from Fluka; cornmeal agar (CMA) from HiMedia Laboratories (Mumbai, India); hydrogen peroxide (H_2_O_2_) from VWR (Fontenay-sous-Bois, France); and RPMI-1640 broth medium (containing L-glutamine and phenol red and lacking bicarbonate) from Biochrom AG (Berlin, Germany). The N-acetylglucosamine–yeast-nitrogen-base–proline (NYP) medium (pH 6.7 ± 0.1) was prepared using GlcNAc (10^−3^ mol/L), YNB (3.35 g/L), proline (10^−3^ mol/L), and NaCl (4.5 g/L). Caffeic acid cornmeal agar (CACA) was prepared by supplementing CMA with CA previously dissolved in PBS (final concentration of 0.3 g/L).

#### 4.1.2. Culture Media and Reagents Used in Mouse Cell Culture Assays

Fetal bovine serum (FBS) from GE Health Care Life Sciences (GE Health Care, Salt Lake, UT, USA); Dulbecco’s modified Eagle medium/F-12 nutrient mixture (Ham) (DMEM/F-12; 1:1) from Gibco (Paisley, UK); and dimethyl sulfoxide (DMSO), thiazolyl blue tetrazolium bromide (MTT), gentamicin, sulphanilamide, H_3_PO_4_, and naphtylethylenediamide from Sigma-Aldrich (St. Louis, MO, USA). The Griess reagent was prepared as a 1:1 solution of 1% *w*/*v* sulphanilamide solution in H_3_PO_4_ (5% *v*/*v*) and naphtylethylenediamide (0.1%) in deionized water. 

#### 4.1.3. Other Reagents

2,2-Diphenyl-1-(2,4,6-trinitrophenyl)hydrazyl (DPPH) and MeOH from Sigma–Aldrich (Steinheim, Germany).

H_4_[H_2_(^4-CF3^PhCH_2_)_2_Cyclam]Cl_4_ (compound **1**) was synthesized according to a previously published procedure [6]. The stock solution of compound **1** was prepared in DMSO and stored at −20 °C. The different concentrations of the compound were prepared in the appropriate medium/buffer for the tests.

### 4.2. Microorganisms and Murine Macrophage Cell Line 

*Candida* reference strains were obtained from the American Type Culture Collection (ATCC, Manassas, USA) (*C. albicans* ATCC 10231 and *C. krusei* ATCC 6258), and *Cryptococcus neoformans* CECT 1078 was obtained from the Colección Espanõla de Cultivos Tipo (CECT). All microorganisms were kept in SDB plus glycerol (20%) at −80 °C. The yeasts were cultured in SDA for 24 h (for *Candida* spp.) or 48 h (for *C. neoformans*) to obtain optimal growth and ensure the purity of the cultures.

RAW 264.7 murine macrophage cells were provided courtesy of Graciliana Lopes from CIIMAR—Interdisciplinary Centre of Marine and Environmental Research.

### 4.3. Effect on Yeast Biofilm Formation

The effect of compound **1** on biofilm formation was evaluated using the crystal violet method according to Merritt et al. [43]. *C. albicans*, *C. krusei*, and *C. neoformans* suspensions (10^6^ colony-forming units (CFU/mL)) were prepared in SDB, placed in 96-well microplates (100 μL), and incubated for 6 h at 37 °C. The plates were washed twice with a 0.85% saline solution, and increasing concentrations of compound **1** (1/2 MIC, MIC, and 2 MIC) prepared in SDB were added to the wells and incubated at 37 °C for 24 h. The yeasts were again washed twice with the saline solution, and 125 μL of 0.1% crystal violet was added to them. The yeast cells were then washed twice with 0.85% saline followed by a 15-minute incubation with crystal violet. After the incubation, the supernatants were rejected, and the 96-well plates were placed in an inverted position and left for 1 h at room temperature to allow them to dry. The crystal violet was dissolved in 95% ethanol (125 μL), and the absorbance was determined using a spectrophotometer at 550 nm. Amphotericin B and DMSO were included as the positive and solvent controls, respectively.

### 4.4. Effect on Catalase Activity

This assay was based on the technique described by Carvalho et al. [44] with modifications. The effect of compound **1** on catalase activity was evaluated after culturing yeasts on SDA for 24 h. Afterwards, a suspension of 0.7 MacFarland was prepared and a 1:100 dilution in RPMI was performed, and the sample was incubated overnight at 36 °C with agitation. The cells were centrifuged (3500 rpm) and washed with PBS, and the concentration was adjusted to 1 MacFarland with the same buffer. In a centrifuge tube, 10 μL of compound **1** was added to 990 μL of yeast suspension, and the sample was incubated in a water bath at 36 °C with agitation for 2 h. After the treatment, the yeasts were added (1:1) to a 3% H_2_O_2_ solution. A score for the visual effervescence observed was made, with (4) corresponding to the maximum effervescence of the positive control (yeasts with DMSO but without compound **1**), (3) corresponding to 75%, (2) corresponding to 50%, and (1) corresponding to 25% of the effervescency of the control. The effect of the antifungal drug fluconazole on the yeast catalase activity was also tested.

### 4.5. Effect on Candida albicans Dimorphic Transition

The effect of compound **1** on *C. albicans* yeast–mycelium transition was studied. Cultures in SDA (24 h) were used to prepare yeast cell suspensions (1.0 ± 0.2 × 10^6^ CFU/mL) in NYP medium that were placed in glass tubes (990 μL), to which solutions of compound **1** were added to achieve 2 MIC, MIC, 1/2 MIC, and 1/4 MIC values [27]. After a 3 h incubation period at 37 °C, 100 cells from the treated and untreated samples were observed with a light microscope. Germ-tube-positive cells were considered when the germinating tube was at least as long as the diameter of the blastospore, and the percentage of germinating cells was determined. The results were obtained from three independent experiments.

### 4.6. Effect on Cryptococcus neoformans Urease

A suspension with a turbidity of 3 McFarland was prepared in NaCl (9 g/L) from a 48 h culture of *C. neoformans*. Two different interference tests with urease activity were carried out using this suspension. In the first test, 50 μL of the suspension was incubated with 200 μL of different concentration values (2 MIC, MIC, 1/2 MIC, and 1/4 MIC) of compound **1** in saline medium. After incubation for 2 h at 37 °C, the microtubes were centrifuged, the supernatants were discarded, and 200 μL of urea medium was added to the pellet. The microtubes were incubated again at 37 °C overnight and, at the end of the incubation, the color of the tubes was visually assessed in comparison with a positive control (yeast without compound). In the second test, 25 μL of yeast suspension was added to 200 μL of urea medium containing different concentrations of compound **1**. The microtubes were incubated overnight at 37 °C, and then the coloration of each was visually assessed in relation to a control, as described above.

In a different experiment, the interference with urease production was evaluated. In this case, 25 μL of *C. neoformans* suspension in saline was added to 500 μL of RPMI medium supplemented with MOPS containing different sub-inhibitory concentrations of compound **1** (1/2 and 1/4 MIC). After a 48 h incubation at 37 °C, the microtubes were centrifuged, the supernatant was discarded, and 25 μL of the pellet was added to 200 μL of urea medium. The activity of the urease enzyme was assessed by observing the color of each of the microtubes when compared to a positive control, as described above.

### 4.7. Effect on Cryptococcus neoformans Melanin Production

This assay was based on the work described by Li et al. [45] with modifications. Cultures with 24 h growth were adjusted to 1 × 10^3^ CFU/mL in SDB and placed in contact with different concentrations of compound **1** for 6 h in a microtube. The cells were centrifuged at 10,000 rpm for 10 min, and 180 μL of the medium was removed. *C. neoformans* was resuspended in the remaining 20 μL and transferred to a 12-well plate containing 2 mL of CACA. The plates were incubated at 35 °C and regularly observed, and after 8 days of incubation, a pigment score was made by taking the value of the maximum pigment production of a positive control as (5) and assigning the other scores in comparison with that.

### 4.8. In Vitro Determination of Antioxidant Capacity by the DPPH Method

The antioxidant activity of the compound was determined by using a stable radical DPPH assay [46]. Briefly, 200 µL of compound **1** dissolved in MeOH was added into a 96-well microplate, and the sample was then proceeded to two-fold serial dilutions starting from 3000 to 1 µg/mL. Then, 100 µL of a 0.2 mM DPPH solution was added in each well. During 30 min of incubation, the microplate was kept covered to minimize evaporation and kept in the dark. Regarding controls, 100 µL of MeOH and 100 µL of 0.2 mM DPPH solution were added to the positive control wells, and to the negative control wells, 200 µL of MeOH was added. This assay was performed in triplicate. Color intensity was evaluated using a spectrophotometer at 545/630 nm. The scavenging activity of free radicals by the compound was calculated with the following expression:

% DPPH scavenging = 100 × [(abs blank) − (abs sample + DPPH)]/[(abs blank)].

### 4.9. Interference with NO Production by RAW264.7 Macrophages

RAW264.7 cells were seeded at a concentration of 1 × 10^6^ cell/mL (200 µL) in a 96-well flat-bottom culture plate and left in a CO_2_ incubator for 2 h at 37 °C to adhere to the wells [47]. The culture medium was then removed, and the cells were treated with equal volumes of LPS (1.5 µg/mL) and compound **1** solutions for 24 h in a CO_2_ incubator at 37 °C [47]. For NO quantification, 100 µL of the supernatants was transferred to another flat-bottom 96-well culture plate, to which 100 µL of the Griess reagent was added. The reaction was allowed to occur protected from light for 10 min at room temperature. The optical density was measured (545/630 nm; STAT FAX 3200, GMI Trusted Laboratory Solutions, Minneapolis, MN, USA), and the effect on nitrite production was calculated as follows:

Inhibition of NO production (% of control) = 100 − [(abs sample-abs blank)/(abs control − abs negative control) × 100]

After the supernatants had been removed for the Griess reaction, the remaining supernatant was discarded for the MTT test to determine cell viability [48]. The cells were incubated for 4 h at 37 °C with an MTT solution (0.2 mg/mL), and the MTT formazan product was solubilized with DMSO for 10 min while shaking the plate [47]. The absorbance was measured at 545/630 nm (STAT FAX 3200), and the formula used to calculate the compound’s cytotoxicity against RAW264.7 macrophages was as follows:

cellular metabolic viability inhibition (% of control) = 100 − (abs sample/abs control × 100).

### 4.10. Statistics

One-way analysis of variance (ANOVA) and Tukey’s HSD multiple comparison post hoc test were applied to the experiments. The statistical analysis of the results was performed using the SPSS software (IBM^®^ version 20.0). Differences were considered statistically significant at * *p* < 0.05, ** *p*< 0.01 and *** *p* < 0.001 

## 5. Conclusions

Following our previous research work that describes the antifungal activity of cyclam-based derivatives, we demonstrated here the ability of H_4_[H_2_(^4-CF3^PhCH_2_)_2_Cyclam]Cl_4_ to interfere with the virulence factors of *C. albicans*, *C. krusei*, and *C. neoformans*. Moreover, H_4_[H_2_(^4-CF3^PhCH_2_)_2_Cyclam]Cl_4_ also inhibits the initiation of biofilm formation. This is a great advantage because virulence factors are not only related to the severity of the disease that the yeast can cause but also to its ability to resist the effect of antifungal compounds used to treat mycoses. We can therefore conclude that H_4_[H_2_(^4-CF3^PhCH_2_)_2_Cyclam]Cl_4_ may have the potential to be used in antifungal therapy, either as a monotherapy or in combination, although studies into its antifungal mechanism of action and its cytotoxicity are still needed, since the cytotoxicity was observed on the murine macrophage cell line RAW 264.7.

Our in vitro stage study may open paths for future works that are mainly dedicated to human *Candida* spp. and *Cryptococcus neoformans* infection management.

## Figures and Tables

**Table 1:** Effect of compound **1** on *Cryptococcus neoformans* melanin production.

Control	1/8 MIC
4.8 ± 0.5	2.0 ± 0.8 **

MIC, minimal inhibitory concentration; maximum pigment production of positive control was scored as 5, and other scores were assigned in comparison with that as follows: 5, maximum pigmentation; 0, no pigmentation. Four independent experiments were performed, and the results are presented as mean ± SD. ** *p* < 0.01.

**Table 2:** Production of nitric oxide (NO) (% of control) by LPS-stimulated RAW264.7 macrophages after treatment with compound **1**.

Concentration (μg/mL)	NO Production (% of Control)
64	67.5 ± 8.6 *
32	79.5 ± 3.7 **
16	90.8 ± 6.8

Three independent experiments were performed, and the results are presented as mean ± SD. * *p* < 0.05; ** *p* < 0.01.

## Data Availability

Data are contained within the article.
